# Correlation of salivary immunoglobulin A with Body Mass Index and fat percentage in overweight/obese children

**DOI:** 10.1590/1678-7757-2018-0088

**Published:** 2018-11-08

**Authors:** Mayra Manoella Perez, Juliana Souza Pessoa, Ana Lídia Ciamponi, Michele Baffi Diniz, Maria Teresa Botti Rodrigues Santos, Heloísa Helena de Oliveira Alves, Renata Gorjão, Renata Oliveira Guaré

**Affiliations:** 1 Universidade Cruzeiro do Sul, Programa de Pós-Graduação em Odontologia, São Paulo, São Paulo, Brasil; 2 Universidade de São Paulo, Faculdade de Odontologia, Departamento de Ortodontia e Odontopediatria, São Paulo, São Paulo, Brasil; 3 Universidade Cruzeiro do Sul, Programa Interdisciplinar de Pós-Graduação em Ciências da Saúde, Instituto de Ciências da Atividade Física e Esporte, São Paulo, São Paulo, Brasil

**Keywords:** Gingivitis, Saliva, Pediatric obesity

## Abstract

Obesity is considered a risk factor for periodontal health due to the low- grade inflammation promoted by the increased adipose tissue. Objective: This study aimed to determine correlations and associations between gingival inflammation (Simplified Oral Hygiene Index, and Gingival Index), salivary immunoglobulin A (s-IgA), and salivary parameters (salivary flow and osmolality) in normal-weight and overweight/obese children. Material and Methods: Ninety-one children, aged 6 to 12 years old (8.6±1.9 years), were divided into two groups according to their body mass index (BMI), circumferences, skinfold measurements and body fat percentage: normal- weight group (NWG; *n* =50) and overweight/obese group (OG; *n* =41). A calibrated examiner performed the clinical examination using the Simplified Oral Hygiene Index, Gingival Index, and salivary collection. Data analysis included descriptive statistics and association tests ( *p* <0.05). Results: OG presented statistically higher s-IgA values compared with NWG, especially among the obese children ( *p* <0.05). Significant positive correlations between s-IgA and salivary osmolality in OG ( *p* <0.05), and between s-IgA and BMI values ( *p* <0.05) and body fat percentage ( *p* <0.05) were observed among all the children. Effect size varied from moderate for s-IgA values ( *d* =0.57) to large for BMI ( *d* =2.60). Conclusion: Gingival inflammation and salivary parameters were similar for NWG and OG; however, s-IgA presented higher values in OG, with correlations between BMI and body fat percentage.

## Introduction

The prevalence of obesity has increased in recent decades and is a public health concern, given that this condition is an important risk factor for the onset of several systemic diseases. ^1^ In addition, obesity can alter the production and release of important defense cells, such as neutrophils, recognized as the first line of defense for periodontal tissues, and T and B lymphocytes, responsible for cellular and humoral responses. ^2^ The inflammatory state is reflected by an increase in circulating levels of proinflammatory proteins and occurs not only in adults but also in children and adolescents. Inflammatory mediators are secreted by the adipose tissue and the immune system, which can lead to a hyperinflammatory state. ^3^ ^,^ ^4^

Most oral inflammatory diseases seem to originate locally, but present predisposing systemic factors. ^5^ Thus, obesity is considered a risk factor for periodontal health. ^6^ Although systematic reviews of the literature have observed a higher prevalence of periodontal disease in obese adults, ^7^ few studies have investigated periodontal health in overweight/obese children and adolescents. ^3^ ^,^ ^8^ Gingivitis is widely prevalent in subjects of all ages, including children and adolescents, and usually precedes periodontitis. ^9^ Thus, general practitioners should be aware of the link between obesity and periodontal health. ^10^ A recent systematic review and meta-analysis suggested periodontal alterations might be associated with obesity in children. ^1^

Salivary immunoglobulins play an important role in inflammatory diseases of the oral mucosa. ^5^ Salivary immunoglobulin A secretion has a protective effect against oral bacteria, inhibiting the adhesion of microorganisms to the surface of mucosal cells, thus preventing their penetration into the organic tissues. ^5^ ^,^ ^11^ Studies have been suggesting an elevated s-IgA level is associated with a lower risk of developing gingivitis; ^12^ however, studies on this association in children remain scarce. In addition, studies have shown chronic stress promotes a decrease in s-IgA in children. ^13^ Therefore, s-IgA is an important biomarker of immune imbalances in children and may be related to oral disease development.

There is a known association between obesity and inflammation, ^8^ and periodontal disease is a rare finding in children. ^10^ Therefore, the purpose of this study was to determine correlations and associations between gingival inflammation, s-IgA, and salivary parameters in normal-weight and overweight/obese children classified according to Body Mass Index (BMI), skinfold measurements, and fat percentage. The hypothesis is that overweight/obese children present greater gingival inflammation and different salivary parameters compared with normal-weight children.

## Materials and methods

### Ethical statement

This study was approved by the local Research Ethics Committee under protocol CE-UCS-031/2012 (Cruzeiro do Sul University).

### Sample selection

The study population consisted of children who sought dental care at a dental school in São Paulo, SP, Brazil, from October 2014 to May 2016. Healthy children of both genders, aged 6 to 12 years old, in the mixed-teething phase were selected. They were divided into two groups: normal weight group (NWG) and an overweight/obese group (OG), based on anthropometric assessments.

The exclusion criteria were: children who had any type of infection, diabetes, and leukemia, as well as asthmatic bronchitis; those who were administered any drug that could interfere with salivary secretion (anticholinergics, neuroleptics, and benzodiazepines) at least 72 h before salivary collection; and those who refused to cooperate with data collection.

Sample size was based on a previous study by Modéer, et al. ^3^ (2011) on obesity and periodontal risk factors in adolescents. This was calculated at a 95% confidence interval and a study power of 80%. The OpenEpi software ( http://www.openepi.com ) was used, and a sample size of 52 children *per* group was calculated.

Short talks were held with the children's parents or guardians to explain the project and the importance of nutritional and dental assessment. Parents/guardians were interviewed to complete a medical history questionnaire, to identify possible health problems. The research was carefully explained to the parents/ guardians of the participants, who signed a term of free, informed consent.

Specific days were scheduled the same week for saliva collection, oral cavity examinations, and bioimpedance, according to availability of the children and their parents/guardians. Children who required dental treatment were attended at the university.

### Body Composition Assessments

Body composition assessments were performed by a trained and calibrated examiner (intraclass correlation coefficient >0.9), who has significant experience in the field (R.G.).

To calculate the BMI (mass/height ^2^ ), height was measured with a portable vertical stadiometer (Personal Sanny^®^, São Bernardo do Campo, SP, Brazil), 2 m in length, accurate to 0.1 cm. Body mass (kg) was measured with an electronic scale, accurate to 100 g. BMI was calculated using the formula: BMI = body weight (kg)/height ^2^ (m). For the nutritional profile, benchmarks in *z score* based on BMI curves for boys and girls proposed by the World Health Organization (WHO) in 2007 ^14^ were used: z score <85 was designated as normal weight, z score of 85 to 97 was designated as overweight, and >97 was designated as obese.

A measuring tape, accurate to 1 mm, was used to determine the following body circumferences: forearm, abdomen, hip, waist, and calf. An adipometer (Sanny^®^, São Bernardo do Campo, SP, Brazil) accurate to 0.5 mm, was used to measure skinfolds. The percentage of fat was calculated using the formula of Slaughter, et al. ^15^ (1988), and was performed by measuring triceps skinfold (TR) and subscapularis skinfold (SS). The formulas were: Body fat (BF)= 0.783x(TR+SS)+1.6 for boys and BF= 0.546x(TR+SS)+9.7 for girls. The standards adopted to classify children were based on the Lohman classification ^16^ ; from 11 to 20% children are classified as eutrophic; from 21 to 25%, as overweight; and above 25%, as obese. The circumference and skinfold measurements were used to confirm the BMI classification for children.

Moreover, body composition (percentage fat, fat body mass, and lean body mass) was estimated using the bioimpedance analysis (BIA) to characterize the sample and detect possible nutritional problems. In the BIA analysis, a low-level electric current is passed through the body of the subject and the impedance (z), or opposition to the current flow, is measured with a BIA analyzer A310 (Biodynamics^®^, Shoreline, WA, USA). The BIA measurement was performed on the right side of the body, with the child lying supine on a non-conductive surface in a room with a normal temperature (~22°C). The volunteers were on an eight-hour fast in the test day. First, the skin was cleaned at the electrode placement points with alcohol. Subsequently, the electrode sensors (proximal) were placed on the dorsal surface of the wrist joint so that the upper edge of the electrode aligned to the head of the ulna, and the dorsal surface of the ankle so that the upper edge of the electrode aligned with the medial and lateral malleoli. The placement of the source electrodes (distal) was at the base of the second or third metacarpophalangeal joint of the hand and the metatarsophalangeal of the foot. The individual's arms and legs were spaced approximately 45° from each other. ^17^

### Assessment of gingival inflammation

Another experienced and calibrated examiner (weighted Kappa >0.8) (R.O.G.) assessed the children's gingival status in a dental office with a reflector light, a triple syringe, a flat mouth mirror, a WHO periodontal probe with 3.5, 5.5, 8.5, and 11.5 mm markings, and sterile gauze. Then, the same examiner calculated the Simplified Oral Hygiene Index (OHI-S) and Gingival Index (GI) coefficients.

The patient's oral hygiene assessment followed the criteria used in the OHI-S proposed by Greene and Vermillion ^18^ (1964): on the buccal surfaces of teeth 11/51, 31/71, 16/55, 26/65, and the lingual surfaces of teeth 36/75 and 46/85. The OHI-S is a combination of plaque indices and calculations, with scores ranging from 0 to 3. The plaque indices and the sum of the assigned scores were calculated separately and then divided by the number of surfaces examined. Dye was not used to obtain this index.

The gingival index, proposed by Löe and Silness ^19^ (1963), was determined for each child based on the same teeth assessed in the OHI-S: 11/51, 31/71, 16/55, 26/65, 36/75, and 46/85. The presence or absence of bleeding was verified by pressure stimulus (probing) against the inserted and papillary gingivae, again scored from 0 to 3. The tissues around each tooth, the distobuccal papilla, vestibular margin, mesiobuccal papilla, and linguogingival margin, were also assessed.

### Assessment of salivary parameters

Samples of unstimulated total saliva were collected from children who had not performed physical exercise for 24 h. Saliva was always collected in the morning, between 8 and 10 am, for 5 min (timed by a stopwatch), to minimize the circadian rhythm effects. The child was seated in a relaxed position, with their head slightly lowered.

Before saliva collection, the mouth was sanitized with distilled water, and the initial saliva was discarded before the stopwatch was used to determine salivary flow (mL/min).

The saliva was collected using a funnel into a 50 mi- conical tube. Immediately after completing collection, the samples were stored on ice and transported in a styrofoam box to the salivary analysis laboratory. Salivary volume and flow (mL/min) were calculated, and then the saliva was centrifuged at 5000 rpm for 5 min (Hettich^®^ centrifuge, Universal 320R model, Tuttlingen, Germany). The samples were stored at −80°C until analysis.

Salivary osmolality was determined after thawing the samples. Ten microliters of centrifuged saliva were placed on the optical reading disk of the osmometer (VAPRO^®^ Vapor Pressure Osmometer, model 56000; New Instrument, Washington, DC, USA). The osmometer was calibrated by the comparison method using standard solutions for salivary osmolality (Opti- Mole^TM^ 290 and 1000 mmol/kg Osmolality Standard ELITech Group, WESCOR, Washington, DC, USA) before readings were taken.

Salivary IgA was determined using the indirect competitive enzyme immunoassay (EIA) method, according to the manufacturer's instructions (SpectraMax Plus^®^, Molecular Devices, CA, USA and Salimetrics^®^ IgA Kits, PA, USA) and the protocol adapted by Sari-Sarraf, et al. ^20^ (2011). A standard curve was generated using known concentrations of IgA. Subsequently, 25 μL of each sample was aspirated, then poured into a microplate together with equal aliquots of the working reagents provided in the kit, according to the manufacturer's specifications. After adding the samples, the staining was analyzed by reading the microplate in a spectrophotometer at a wavelength of 450 nm. The results were expressed as μg/mL.

### Statistical analysis

Data analyses included descriptive statistics and association tests. Subsequently, groups were compared to verify the homogeneity in relation to the characteristics studied. Normality assumption and homogeneity of variances were evaluated using the Kolmogorov-Smirnov and Levene tests, respectively.

The body composition, salivary composition, and gingival inflammation variables for the two groups were compared by Student's *t* -test (parametric data) and the Mann-Whitney test (nonparametric data). The magnitude of the statistical difference was assessed by determining the effect size (ES). ^21^ The classification of ES was as follows: ≤0.2 indicated a small effect, 0.30.7 indicated a moderate effect and ≥ 0.8 indicated a large effect. Correlations between the variables were verified by the Spearman correlation coefficient (nonparametric data). The level of significance was set at 5%.

In addition, analysis of the s-IgA data, considering the normal-weight, overweight, and obese children separately, was performed using the Kruskal-Wallis and Bonferroni *post hoc* tests. Finally, a linear regression was performed to predict IgA levels, using BMI as the main predictor, adjusting for gingival inflammation, age, and gender. All statistical analyses were performed using IBM SPSS software version 20.0.

## Results

The final study sample consisted of 91 children of both sexes, 50 normal-weight children and 41 overweight/obese children, aged 6 to 12 years old (8.6±1.9 years). In the OG, 16 children were overweight and 25 were obese. During data collection, the NWG had a sample loss of two children, while the OG lost eleven children, who were unable to attend every stage of the research.

Children in the NWG ( *n* =50) had a mean age of 8.1±1.7 years old; 26 (52%) were female and 24 (48%) were male. Children in the OG ( *n* =41) had a mean age of 8.6±1.8 years old; 27 (65.8%) were female and 14 (34.2%) were male. The sample was homogeneous in terms of age (Mann-Whitney test, p=0.185) and sex (Chi-square test, p=0.182).

Statistically significant differences were observed between the groups regarding body composition and salivary variables (BMI, mean skinfold measurement, fat percentage, and s-IgA values), nonparametric values were higher in OG ( *p* <0.05) ( Table 1 ). ES estimates were large for BMI ( *d* =2.60), fat percentage ( *d* =2.10) and mean skinfold measurement ( *d* =1.70), and moderate for s-IgA values ( *d* =0.57).

**Table 1:** Body composition, salivary composition and gingival inflammation of normal-weight and overweight/obese children

Variable	Mean ± SD		p-value	effect size (d)
	NWG	OG		
BMI (Kg/m^2^)	16.11±1.65^A^	22.81±3.26^B^	<0.001 (1) *	2.6
Fat %	19.52±5.90^A^	30.25±4.17^B^	<0.001 (2) *	2.1
Mean skinfold (cm)	18.38±5.45^A^	29.64±7.66^B^	<0.001 (1) *	1.70
s-IgA μg/mL)	155.46±122.04^A^	247.32±190.40^B^	0.029 (1) *	0.57
Osmolality (mOsm/Kg H_2_O)	68.04±19.91^A^	74.71±18.41^A^	0.104 (2)	0.35
Flow (mL/min)	0.67±0.38^A^	0.65±0.31^A^	0.933 (1)	0.06
OHI-S	1.17±0.62^A^	1.01±0.54^A^	0.317 (1)	0.28
GI	1.30±0.48^A^	1.29±0.54A^A^	0.836 (1)	0.02

Note: NWG, normal-weight group; OG, overweight/obese group; OHI-S, Simplified Oral Hygiene Index, GI, gingival index. Different uppercase letters indicate a statistically significant difference on the same line.

(1) Mann-Whitney test;

(2) Student t test;

* p<0.05

**Figure 1:**
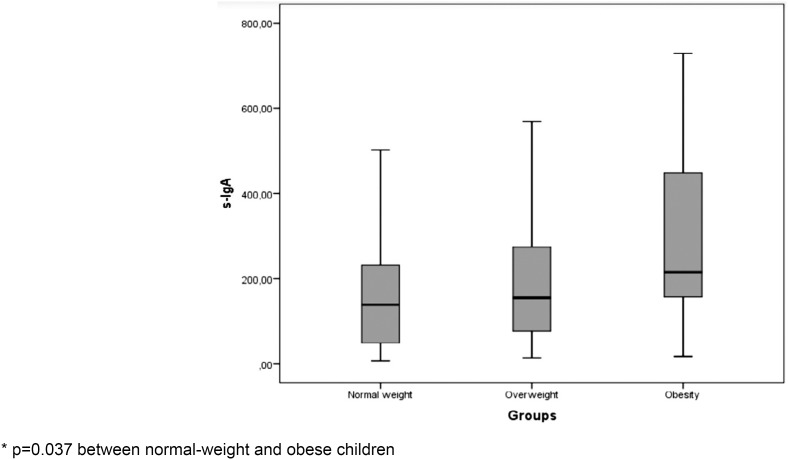
Salivary IgA concentrations according to the classification of weight status.

In addition, salivary IgA levels were compared for normal-weight, overweight, and obese children, and a significant difference was observed between normal- weight and obese children ( *p* =0.037), demonstrating that s-IgA levels increase as weight status classification increases ( Figure 1 ).

The correlation between s-IgA levels and parameters related to obesity (BMI and fat %) among all the children in the sample was significantly positive between s-IgA and BMI values, and s-IgA and fat percentage values ( *p* <0.05) ( Table 2 ).

**Table 2:** Spearman coefficient of correlation between s-IgA and body composition variables in the children studied

Variable	Spearman's rho	p-value
IgA-s x BMI	0.287	0.006 *
IgA-s x Fat %	0.266	0.011 *
IgA-s x mean skinfold %	0.087	0.410

Note:

* Correlation is significant at p<0.05

Correlations were tested between salivary parameters (salivary flow, osmolality, and s-IgA) and gingival inflammation (OHI-S and GI) in the NWG and OG. No significant correlations were verified in the NWG between the variables investigated ( *p* >0.05). However, a significant correlation was verified between s-IgA and salivary osmolality in the OG ( *p* <0.05)( Table 3 ).

**Table 3:** Spearman coefficient of correlation between salivary variables (salivary flow, osmolality, s-IgA) and gingival inflammation in normal-weight and overweight/obese children

Normal-weight group						
Variable		Flow	Osmolality	IgA-s	OHI-S	GI
Flow	Spearman's rho	1				
	p-value					
Osmolality	Spearman's rho	-0.189	1			
	p-value	0.188				
IgA-s	Spearman's rho	-0.232	0.202	1		
	p-value	0.104	0.160			
OHI-S	Spearman's rho	0.050	-0.098	-0.137	1	
	p-value	0.731	0.498	0.341		
GI	Spearman's rho	-0.073	0.011	-0.233	0.436 **	1
	p-value	0.614	0.942	0.104	0.002	
**Overweight/obese group**						
**Variable**		Flow	Osmolality	IgA-s	OHI-S	GI
Flow	Spearman's rho	1				
	p-value					
Osmolality	Spearman's rho	-0.197	1			
	p-value	0.216				
IgA-s	Spearman's rho	-0.157	0.326 *	1		
	p-value	0.328	0.038			
OHI-S	Spearman's rho	-0.174	0.157	-0.071	1	
	p-value	0.278	0.327	0.660		
GI	Spearman's rho	-0.240	0.160	-0.268	0.455 **	1
	p-value	0.131	0.317	0.091	0.003	

Note: Simplified Oral Hygiene Index, GI, Gingival Index.

** Correlation is significant at p<0.01;

* Correlation is significant at p<0.05

The results of linear regression analysis revealed that BMI (B=12.98; SE=4.07; p=0.002) remained as a significant predictor of s-IgA after adjusting the model for gingival inflammation, age, and gender ( Table 4 ).

**Table 4:** Results of linear regression analysis to predict S-IgA levels, considering body mass index as the main predictor

Coefficients			
Model(a)	B	SE	p-value
(Constant)	20.39	110.59	
BMI	12.98	4.07	0.002 *

Note: R2=0.183;

* p<0.05;

(a) Model adjusted for gingival inflammation, age, and gender

## Discussion

This investigation observed overweight/obese children showed higher s-IgA values, with correlations between BMI and body fat percentage. Thus, confirming the study the hypothesis that obesity can influence immunological and inflammatory processes. ^3^

It is known that salivary proteins are a more sensitive and specific indicator for certain oral diseases. Although our study did not evaluate IgA in plasma, other authors have assessed this condition ^22^ in plasma and saliva, and have reported a good relationship between these variables. Thus, saliva collection is a valid, non-invasive method that is easy to perform and requires less pre-analysis handling, and as reduces the pain and anxiety that is typically associated with blood tests. ^23^

To our knowledge, this is the first study to determine correlations and associations between gingival inflammation in children classified according to BMI, skinfold measurements, and body fat percentage. Most of epidemiological studies use BMI as a method to assess excess body fat because it is easily applied during surveys. ^24^ The z score based on BMI curves for boys and girls proposed by WHO in 2007 ^14^ is the main measurement used to determine overweight and obesity in children. However, the accuracy of BMI in defining obesity is questionable, as it does not distinguish between adipose mass and muscle mass. Thus, the abdominal circumference measurements and skinfolds in association with BMI are recommended to eliminate the inconsistencies in the latter, thus improving the assessment of overweight and obesity in the children studied. ^24^

Surveys in different parts of the world have reported that gingivitis is prevalent in children and adolescents. ^9^ Gingival status should be accurately defined, to determine and control risk factors when assessing these data. Several gingival indices have been proposed in the literature on gingival inflammation in children and young adults. ^25^ The GI was used in this investigation because it has good sensitivity and reproducibility and provides optimal knowledge of periodontal biology and pathology for the examiners. Moreover, this index [proposed by Löe and Silness ^19^ (1963)] has been used in previous studies involving young children. ^26^ ^,^ ^27^ GI may be scored for the all surfaces of all the teeth or the selected teeth or for selected areas of all the teeth or the selected teeth. ^25^ In this study, GI was performed on six index teeth, as suggested by a clinical protocol in the periodontal management of children and adolescents. ^28^ Gingival status evaluation of all surfaces and teeth in young children is very difficult due to behavioral aspects.

A recent systematic review ^1^ suggested a significant positive association between periodontal disease and obesity in children. In this study, the amount of biofilm (assessed by OHI-S) and inflammation (assessed by GI) were similar between the two groups. These results disagree with the consensus in the literature, which shows individuals with obesity have more gingivitis and more biofilm than the respective control group. ^3^ ^,^ ^27^ We believe that the differences between the literature and our results could be explained by the methodology of periodontal assessment (periodontal pocket probing and bone loss) and the age range of the individuals studied (10 to 17 years old).

The reduction in salivary flow is considered an important risk factor for periodontal diseases. ^29^ However, the salivary flow herein was considered normal, and no significant differences were observed between the groups. A previous study verified a correlation between salivary osmolality (which reflects the individual's hydration status) and gingival inflammation. ^29^ The authors reported that increased osmolality reduced salivary flow by increasing molecular cohesion, leading to increased risk of development of gingivitis. ^30^

A cross-sectional study demonstrated that individuals with obesity had significantly more gingival bleeding than the controls in adolescents. ^6^ In our study, although no differences were observed in gingival status among children of either group, the s-IgA values were statistically higher for the OG.

Moreover, a significant positive correlation between s-IgA and parameters related to obesity (BMI and fat %) and linear regression analysis confirmed BMI as the main predictor of s-IgA levels for all of the children studied. However, NWG and OG showed no significant correlation between s-IgA and GI. Salivary IgA is responsible for the immune response in the oral cavity, inhibiting the adhesion of gram-negative bacteria to the epithelium, neutralizing certain bacterial toxins and acting as a defense against microorganisms entering the gastrointestinal and respiratory tracts. ^31^

A nonsignificant negative correlation was observed between s-IgA and salivary flow for both groups. In contrast, a significant negative correlation was observed between salivary flow rate and IgA levels in healthy adults. ^32^ A moderate positive association was observed between salivary osmolality and s-IgA in the OG group. This suggests the higher s-IgA levels in this group may influence salivary viscosity since obese individuals present a higher concentration of salivary proteins compared with normal-weight individuals. ^33^

The study shows some limitations, including the study design (cross-sectional), the convenience sample, the age range of the participants, certain difficulties in maintaining the adhesion and involvement of the parents/guardians of the participating children, and evaluation of only six index teeth for GI instead of all the surfaces. Thus, this study shows internal validity. Although the current literature shows an association between periodontal disease and obesity, longitudinal and experimental studies in different populations are required to determine whether obesity is a risk factor for this disease, particularly among children and adolescents.

Analysis of the results herein leads us to conclude that gingival and salivary inflammation were similar in normal-weight and overweight/obese children. Only s-IgA presented higher values in overweight/obese children and showed correlations between BMI and fat percentage.
